# *Plasmodium vivax* Landscape in Brazil: Scenario and Challenges

**DOI:** 10.4269/ajtmh.16-0204

**Published:** 2016-12-28

**Authors:** Andre M. Siqueira, Oscar Mesones-Lapouble, Paola Marchesini, Vanderson de Souza Sampaio, Patricia Brasil, Pedro L. Tauil, Cor Jesus Fontes, Fabio T. M. Costa, Cláudio Tadeu Daniel-Ribeiro, Marcus V. G. Lacerda, Camila P. Damasceno, Ana Carolina S. Santelli

**Affiliations:** 1 Instituto Nacional de Infectologia Evandro Chagas, Fundação Oswaldo Cruz (Fiocruz), Rio de Janeiro, Brazil.; 2 Programa de Pós-Graduação em Medicina Tropical, Universidade do Estado do Amazonas, Manaus, Brazil.; 3 Pan American Health Organization, Brasilia, Brazil.; 4 Coordenação Geral do Programa Nacional de Controle da Malaria, Ministério da Saúde, Brasilia, Brazil.; 5 Fundação de Vigilância em Saúde, Manaus, Brazil.; 6 Núcleo de Medicina Tropical, Universidade de Brasília, Brasilia, Brazil.; 7 Universidade Federal do Mato Grosso, Cuiabá, Brazil.; 8 Universidade Estadual de Campinas, Campinas, Brazil.; 9 Instituto Oswaldo Cruz, Fundação Oswaldo Cruz (Fiocruz), Rio de Janeiro, Brazil.; 10 Fundação de Medicina Tropical Dr. Heitor Vieira Dourado, Manaus, Brazil.; 11 Instituto Leônidas e Maria Deane, Fundação Oswaldo Cruz (Fiocruz), Manaus, Brazil.

## Abstract

Brazil is the largest country of Latin America, with a considerable portion of its territoritory within the malaria-endemic Amazon region in the North. Furthermore, a considerable portion of its territory is located within the Amazon region in the north. As a result, Brazil has reported half of the total malaria cases in the Americas in the last four decades. Recent progress in malaria control has been accompanied by an increasing proportion of *Plasmodium vivax*, underscoring a need for a better understanding of management and control of this species and associated challenges. Among these challenges, the contribution of vivax malaria relapses, earlier production of gametocytes (compared with *Plasmodium falciparum*), inexistent methods to diagnose hypnozoite carriers, and decreasing efficacy of available antimalarials need to be addressed. Innovative tools, strategies, and technologies are needed to achieve further progress toward sustainable malaria elimination. Further difficulties also arise from dealing with the inherent socioeconomic and environmental particularities of the Amazon region and its dynamic changes.

## Background

Brazil is the largest country in South America, with an area of more than 8.5 million square kilometers and a total population surpassing 200 million inhabitants. With much of its territory lying within the tropical zone (of which almost 60% is in the Amazon region) and a highly diverse flora and fauna, there is a remarkable high receptivity to mosquito-borne infections such as yellow fever, dengue, chikungunya, Zika virus, and malaria.^1^ Malaria transmission is almost entirely restricted to the Amazon region. For the last four decades, Brazil has been responsible for over 30% of the malaria cases in the Americas; in 2014, Brazil reported around 37% of the cases in the American continent.^2^

The history of malaria control in Brazil comprises relevant successes as well as major challenges and barriers.^3^ Until the first half of the 20th century, malaria transmission occurred within most of its territory, with an estimated 6 million episodes per year in a population of 50 million inhabitants. The strengthening of malaria control actions during the Global Malaria Eradication Campaign in the 1950–1960s achieved transmission interruption in the south and northeastern regions of the country.^3^ In the late 1960s, malaria incidence fell below 53,000 cases per year, with almost all of them in the northern region.^4^ Vastly covered by the Amazon rainforest, this territory was characterized by very low population densities and limited mobility between scattered villages, explaining both the low number of cases and even lower presumed fatality rate, attributed to acquired immunity of those populations during that period.^4^,^5^

**Figure 1.**
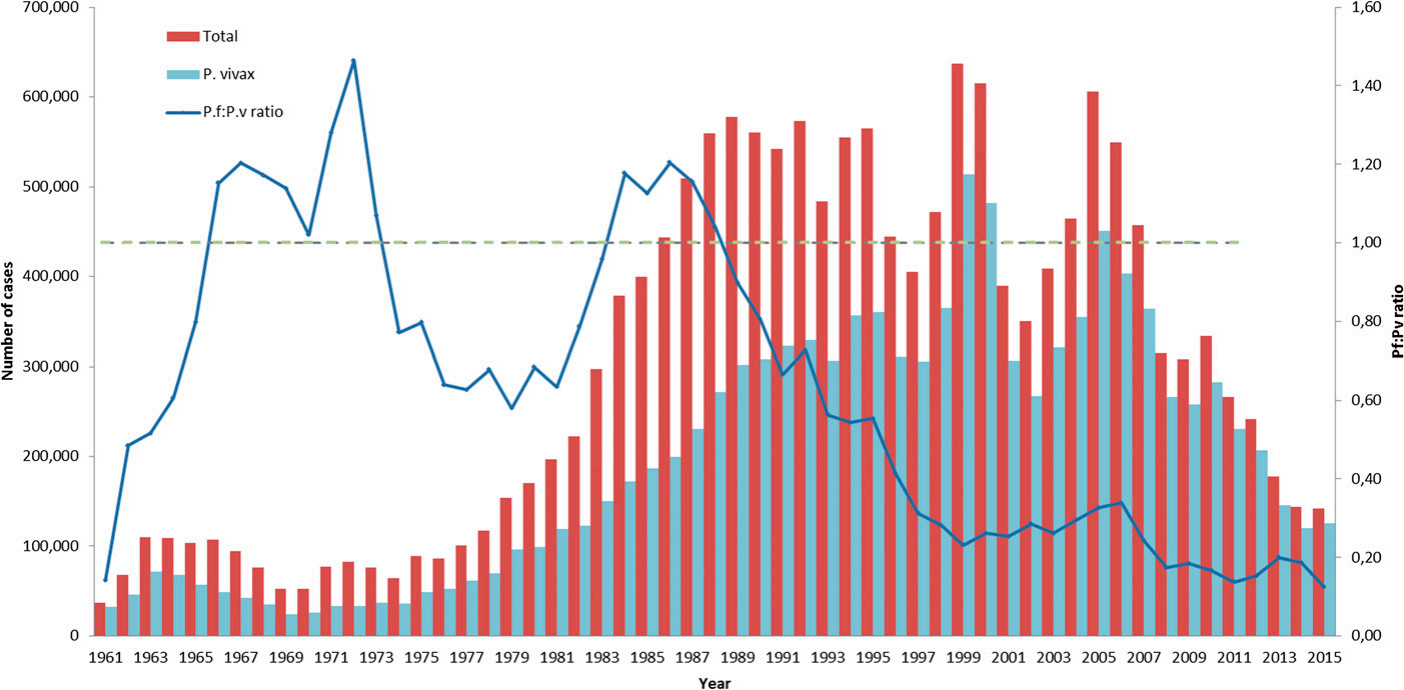
Malaria incidence in Brazil. Absolute number of malaria cases in Brazil in the 1960–2015 period are shown as total cases (red bars) and due to *Plasmodium vivax* (pale blue bars) corresponding to the left *y* axis. The *Plasmodium falciparum*:*P. vivax* ratio is shown as a blue line (right *y* axis, Pf:Pv ratio).

**Figure 2.**
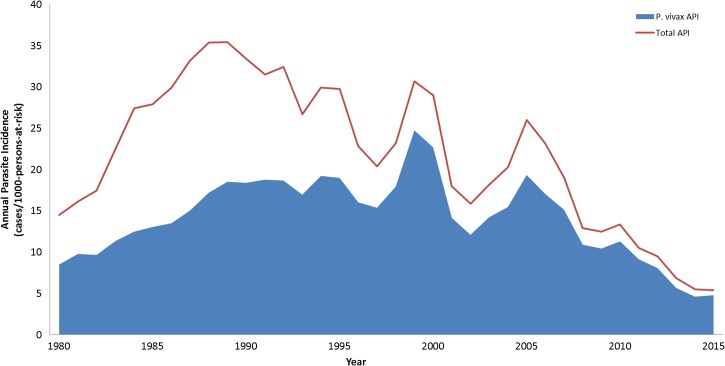
Annual parasite index (API) in Brazil between 1980 and 2015 is shown (red line), as well as API for *Plasmodium vivax* (blue).

However, this scenario began changing in the 1970s when two initiatives of the federal government—1) the creation of a tax-free industrial park in the city of Manaus, located in the western Amazon region; and 2) incentives to cattle and agricultural colonization—led to a massive influx of people to the Amazon region.^5^ The combination of a nonimmune migrant population, inadequate housing conditions, and a vector-abundant environment provided conditions for a rapid and sustained upsurge in the number of malaria cases which only more recently started showing stable signs of reduction, both in absolute numbers (Figure 1

) and in annual parasite incidence (Figure 2

).

Malaria transmission in Brazil is greatly affected by sociodemographic, political, and environmental particularities of the Amazon region. Although there has been a reduction in the absolute incidence of malaria in the country, an increasing proportion of cases are caused by *Plasmodium vivax*, which has become the predominating malaria-causing species in Brazil.^3^ Its distinct characteristics, along with an incomplete understanding of this species' clinical and epidemiological consequences create even greater challenges for transmission control. There is an urgent need for innovative strategies, tools, and technologies to be developed and implemented by scientists and policy makers, especially in a scenario of rapid socioeconomic, political, and environmental changes.^4^,^6^ Herein, we aim to describe and discuss the dynamic landscape of malaria epidemiology in Brazil, focusing on the challenges for controlling *P. vivax* using both national and regional data.

## *Plasmodium vivax* Epidemiology

### Malaria incidence and species proportion.

Malaria incidence has fluctuated in Brazil, with a clear rising trend starting in the late 1970s (Figures 1 and 2). The low number of cases reported in the 1960s was restricted to small villages; this scenario changed in the late 1970s due to a massive migration into the Amazon region promoted by the Federal Government during the military dictatorship.^4^,^5^

The number of cases increased from as low as 52,000 per year in the 1960s to 550,000 in the 1980–1990s, peaking at 637,470 in 1999, followed by a decreasing trend starting in 2008, reaching the lowest number of cases in the last 35 years in 2015, with a total of 142,314 (Figure 1). This trend was also shown by annual parasite index (API) restricted to transmission areas in the Amazon region, which fell from 35 cases per 1,000 persons per year in the early 1990s to slightly above five in 2015 (Figure 2). The decrease in malaria incidence in recent years is a result of efficient control efforts implemented in part due to considerable advocacy by the Roll Back Malaria Initiative reinforced by the Federal Government with support of local innitiatives.^4^

However, despite the relative success of control efforts, malaria transmission is still ongoing in the Amazon region and national-level outbreaks were registered in 1999 and 2005, respectively. A variety of factors can contribute to an upsurge including environmental ones, a lack of appropriate sanitary conditions, and unstable socioeconomic and political scenarios. Specifically, lack of funding and management related to control measures, decentralization of the responsibility for conducting surveillance from the federal to the municipality level, and a more intense rainy season are believed to explain the high incidence observed in 2005. A reduction in malaria incidence leading to discontinuation of control efforts and resources dedicated to malaria control have been also described in other areas.^7^

A steady decline in the proportion of cases due to *Plasmodium falciparum* starting in 1990 with a simultaneous progressive increase in *P. vivax* led to the latter species becoming predominant, and responsible for over 90% of malaria episodes in 2011. Reasons behind this trend, apart from explanations offered in the Biology and Epidemiology reviews in this supplement, may include some important public health landmarks. The creation of the National Universal Health System (SUS) in 1988, based on free, universal health care, resulted in improved access to diagnosis and treatment. *Plasmodium vivax* proportion increased from slightly above 50% on 1983 to around 65% in 1995. During the second half of the 1990s, the Ministry of Health implemented the Intensified Malaria Control Program, expanding and improving diagnosis and treatment of malaria with a focus on reducing time to treatment resulting in around 50% of cases treated within 48 hours of symptoms. The proportion of *P. vivax* reached 80% of the total in 1999, with an increase in the overall number of cases in that year.^3^,^4^

During the following 6 years, the number of cases decreased considerably with a decreasing proportion of *P. vivax* reaching 73% of the total in 2005. This coincided with the decision of the National Malaria Control Program (NMCP) to change the first-line treatment of *P. falciparum* to artemisinin-based combinantion therapies (ACTs) following evidence of increasing resistance of this species to quinine and doxycycline.^8^ Between 2005 and 2015, although the population at risk increased by 13%, malaria incidence decreased considerably as evidenced by reductions in API (79.2%) and total number of cases (76.5%). This trend was accompanied by an even more pronounced decline in the proportion of *P. falciparum*, with *P. vivax* predominance progressively increasing to reach 88.4% in 2015.

A recent study evaluated the epidemiology impact of adopting artesunate–mefloquine fixed-dose combination (ASMQ) for *P. falciparum* in a specific area of the southwestern Amazon.^9^ There was a substantial decrease in total malaria incidence, *P. falciparum*:*P. vivax* ratio and hospital admission rates. One interesting observation was the loss of the characteristic seasonal peak of transmission, which could result from an increasing burden imposed by *P. vivax* relapses, although this was not further investigated.^9^ Additional factors possibly contributing to the relative increase of *P. vivax* need to be considered. For example, due to the longer time for gametocyte production required by *P. falciparum*, enhanced case detection and prompt treatment (especially if initiated up to 72 hours after the onset of symptoms) is considerably more effective at controlling *P. falciparum* than *P. vivax*.^10^ This strategy is more likely to have a high impact in urban areas due to better-established health systems.

### Sociodemographic and environmental influences.

The Amazon's vast territory, largely composed by rainforest intersected by numerous rivers with scattered cities and villages, often experiences environmental changes as well as human occupation patterns influencing malaria transmission. Since 2003, information and surveillance systems have been used to obtain more detailed assessments of malaria epidemiology. More than 60% of transmission occurred in rural areas, with an impressive reduction of urban malaria corresponding to less than 15% of cases since 2010.

During this period, malaria transmission in indigenous areas had increased, contributing to increased mobility of some groups and specific cultural and logistic challenges of providing appropriate health care and controlling transmission for this population, which involve a specific health-care subsystem.^11^ As a result of strategic partnerships between the health sector and the indigenous authorities, including the provision of medical doctors to the indigenous communities through the “Mais Médicos” program,^12^ recent improvement in this area has been reported as a result of better logistics, increased resources leading to more opportune diagnostics and treatment, and the distribution of long-lasting insecticide treated nets (LLINs).

The upward trend for *P. vivax* proportion was shown for all ecotypes from 2005 until 2010, reaching a plateau at around 90% that persisted in following years. Although a higher proportion of *P. vivax* cases were detected in the urban areas, remaining above 90% between 2008 and 2013 (94%), there was a decrease of this proportion to 88% in 2014, with a recovery in 2015 to 91%. Reasons for the drop observed in 2014 can be related to a longer time to diagnose and treat malaria episodes, which could be related to the dissemination of dengue and other arboviral infections to these areas during this period,^13^ which still needs to be properly investigated. A similar pattern was observed for rural and indigenous areas, although for the latter, it only occurred between 2012 and 2015. These differences are consistent with the proposition that better transmission control leads to lower *P. falciparum*:*P. vivax* ratios, therefore reflecting the rapid and stable reduction of cases in the urban settings as compared with a lower and more heterogeneous rate of reduction in the rural and indigenous areas.

**Figure 3.**
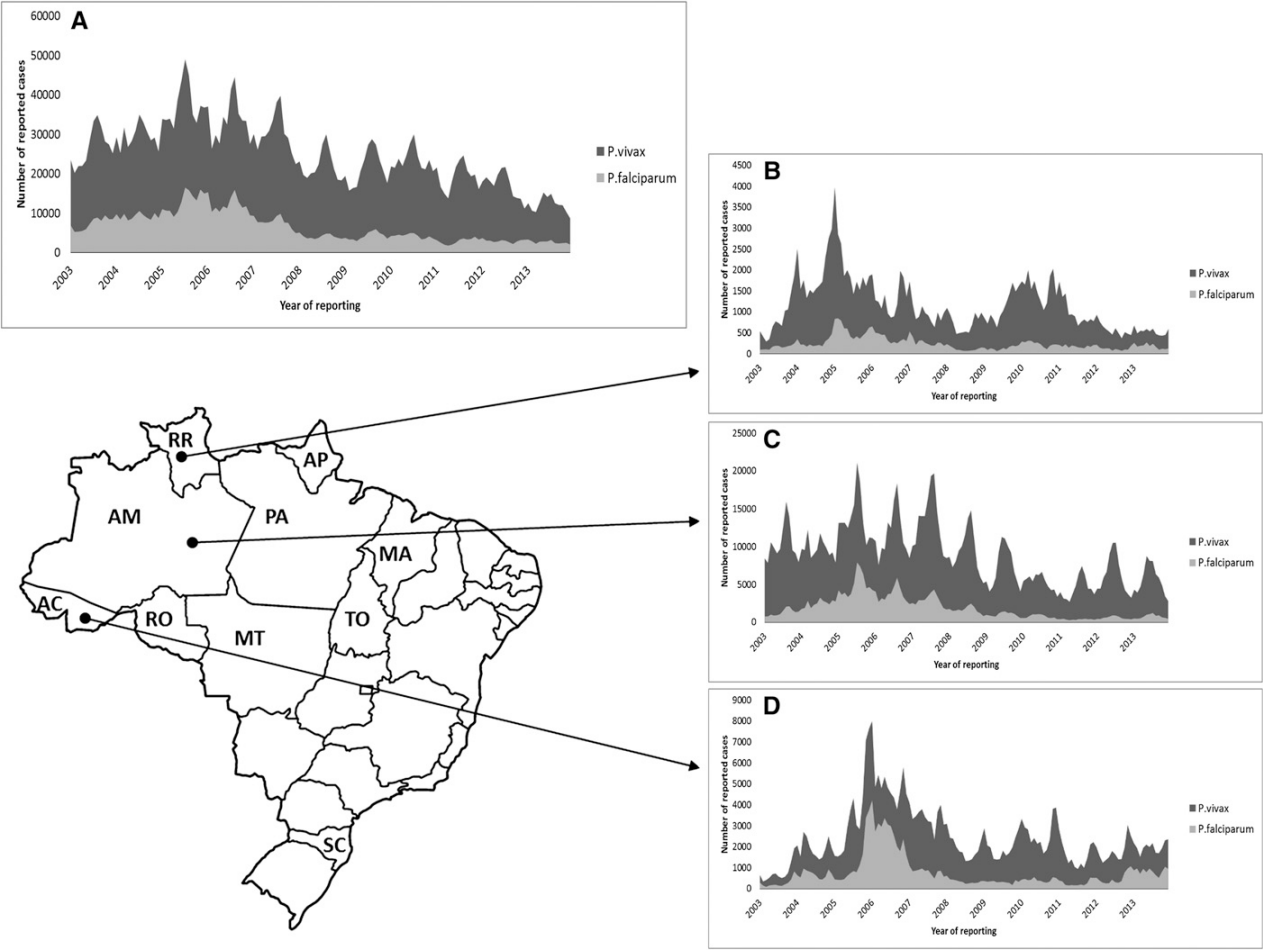
(**A**) Monthly time-series of malaria episodes of *Plasmodium vivax* (light-grey) and *Plasmodium falciparum* (dark-grey) in Brazil, and selected states: (**B**) Roraima, (**C**) Amazonas, and (**D**) Acre. Acronyms in the map correspond to each state in the north region: AC = Acre; AM = Amazonas; AP = Amapa; MT = Mato Grosso; MA = Maranhao; PA = Para; RO = Rondonia; RR = Roraima; TO = Tocantins. SC corresponds to Santa Catarina State in the south region.

The marked seasonality of malaria transmission correlates with the rainy season, explaining the variations observed between different subregions of the Amazon (Figure 3

). This is due to the vector population density. The most important vector is *Anopheles darlingi*, which peaks at the beginning of the dry season, with a minor peak occurring in the beginning of the rainy season.^14^,^15^ This species has a high vectorial capacity with high anthropophilic profile.^14^,^16^ Nontraditional vector species infected with *P. vivax* have been identified in the western Amazon, such as *Anopheles braziliensis* and *Anopheles nuneztovari*.^17^ Although the vectorial potential of these species is probably lower than that of *An. darlingi*,^18^ these findings support an urgent need to better understand how these mosquito-related changes affect malaria transmission.

Hydroelectric dams and gas mining fields lead to important changes affecting the sociodemographic and environmental landscapes and altering malaria transmission dynamics. In the area of the Santo Antonio Dam, near the city of Porto Velho in the southwestern Amazon region, *An. darlingi* was the main vector between 2010 and 2011, with a density peak occurring simultaneously with increasing dam water levels.^19^,^20^ Migration of workers and families into the area combined with increasing mosquito density led to an upsurge on malaria incidence, mainly caused by *P. vivax*.^19^,^21^ Discontinued incentives to building fish farms in some areas had the adverse effect, as many of these ended up abandoned leading to perennial mosquito breeding sites altering the seasonality of malaria as demonstrated in the municipality of Cruzeiro do Sul.^22^ Sophisticated geostatistical methods have also shown an association between fish farming pond locations and endemic and epidemic malaria transmission.^23^ All these introduced elements affecting malaria transmission need to be addressed by specific actions and policies.

The malaria incidence male-to-female ratio has remained unchanged between 2003 and 2015 at 2:1, supporting a work-based risk. The malaria API according to age group decreased for all ages in the same period. Interestingly, the highest burden of disease is reported among the youngest age groups, which suffer proportionally more of *P. vivax* than older individuals. This may be a reflection of both particular transmission patterns and a faster acquisition of immunity against this species compared with *P. falciparum,* as suggested by previous studies.^24^ Younger individuals have been shown to suffer more from anemia when infected, with more pronounced hemoglobin reductions.^25^

### The contribution of relapses.

Recurring *P. vivax* episodes can arise from three sources: 1) reinfections in areas of active transmission; 2) recrudescence as a result of a lack of antischizontocidal efficacy of antimalarials; or 3) relapses, which are the result of the activation of hypnozoites in the liver.^26^ Each of these requires specific control strategies, such as vector management, most efficacious antischizontocidal drugs and antirelapse treatment, respectively. However, there are currently no tools to distinguish between these three sources, which is considered a major hindrance to the design of tailored control actions depending on the epidemiology characteristics of a given area.^26^,^27^

A study in Porto Velho reported in 2010 addressed this issue by counting the number of repeated episodes within a household. Although reinfection could not be ruled out, authors suggested that relapses could account for as high as 30% of malaria episodes in the region.^28^ This finding was similar to a more recent study conducted in a rural settlement in the municipality of Careiro, in the western Amazon, where a rapid decline in malaria incidence occurred between 2008 and 2011.^29^ Although the overall proportion of recurring episodes within 90 days was 29.4%, this rose to 50% in the low-incidence period, suggesting that as vector control measures are successful, the contribution of relapses to the disease epidemiology increases.^29^ Using routine surveillance data, the number of *P. vivax* episodes per person per year was evaluated for 2011 to show that from 23,365 reported episodes at least 23% could be classified as possible relapses. In this study, the number of episodes per person varied from one to more than seven,^30^ despite regular prescription of primaquine. The median time reported from the first episode to first recurrence was 65 days, longer than expected, and the interval to subsequent episodes tended to decrease progressively.^30^ However, the NMCP operational definition classifies episodes occurring within 60 days as relapses, leading to possible missed relapses that occur with longer intervals as well as to inappropriate classification as reinfection and recrudescence can also be an important cause of repeated episodes within this interval range.

Migrants and travelers followed in nonendemic reference centers provide relevant and more unbiased information on vivax malaria relapses, although primaquine use can confound some of the findings. In a study undertaken in Sao Paulo, one-third of *P. vivax*–infected patients treated with chloroquine and primaquine (15 mg/day for 14 days) presented relapses between 1 to more than 6 months after the initial episode.^31^ A recent series of patients followed up in a reference center in Rio de Janeiro described 39.6% of patients presenting relapse, where receiving a total primaquine dose below 3.5 mg/kg (administered during 14 days) was the most important factor associated with recurrence.^32^ Six individuals who acquired *P. vivax* in the Brazilian Amazon region presented incubation periods longer than 3 months,^33^ with important implications for control of this parasite in an elimination context. Molecular studies suggest that relapses in the region are usually the result of multiclone activation of hypnozoites,^34^ as observed also in southeast Asia.^35^,^36^

### Malaria hospitalization and deaths.

**Figure 4.**
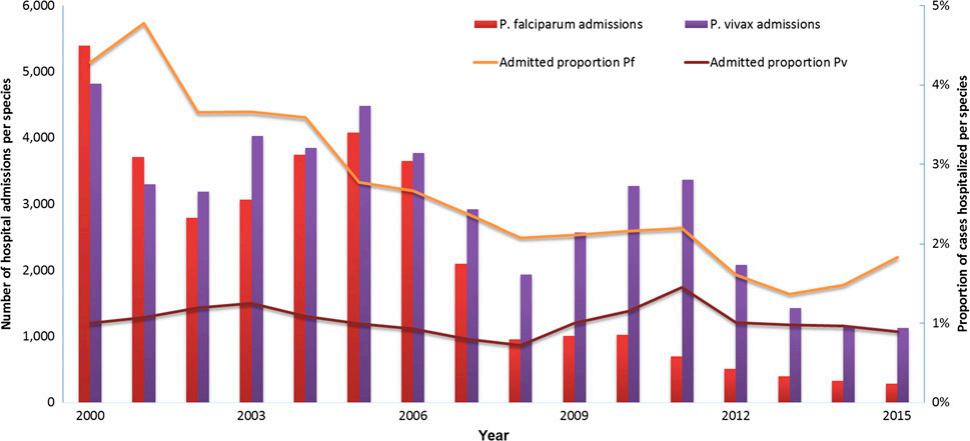
*Plasmodium falciparum* and *Plasmodium vivax* hospital admissions in the period from 2000 to 2015. The left *y* axis shows number of hospital admissions corresponding to *P. falciparum* infections (red bars) or *P. vivax* (purple bars). The right *y* axis shows percent of hospital admissions due to *P. falciparum* infections (yellow line) or *P. vivax* infections (brown line).

*Plasmodium falciparum* hospital admissions decreased from 5,396 in 2000 to 697 in 2012, further dropping to 288 in 2015 (Figure 4

). A reduction in malaria incidence as well as in hospital admissions, which went from slightly below 5% in 2000–2001 to slightly above 2% in 2008, and around 1.5% between 2012 and 2015, attributed to the result of health system improvements in the region (Figure 4). The *P. vivax* admission rate remained low around 1% from 2000 to 2006, further decreasing to 0.73% in 2007 and 2008; however, it increased from 2009 reaching a peak of 1.5% in 2011. It then dropped to 0.98% and 0.9% in 2013 and 2015, respectively. In a restricted setting, this same trend had been reported in 2007 in a tertiary center in Manaus.^37^ Reasons for this change are not clear and could be related to infection-related complications, as *P. vivax* is recognizably associated with severe disease,^38^,^39^ concurrent infections,^40^ or treatment-related complications, as primaquine is given without assessment of glucose-6-phosphate dehydrogenase (G6PD) deficiency status, a topic that is currently under investigation.

The number of malaria deaths decreased from 245 in 2000 to only 39 in 2015 Mortality in the Amazon region; however, it is 100-fold lower (0.017%) compared with non-Amazon regions (1.8%), a difference that could be due to delays in diagnosis and to a higher proportion of imported *P. falciparum*.^41^

Although the malaria surveillance system registry (SIVEP_Malaria) provides reliable case report data, there is no communication with the national mortality registry (SIM), resulting in 40% of reported deaths due to malaria lacking parasite species information. As an example, among the 39 deaths in 2011 with species information, two-thirds were due to *P. vivax* infection. The possibility of underreporting cannot be underestimated, as it could be a contributing factor not reported by the attending physicians. An autopsy study of *P. vivax–*related deaths demonstrated that this infection could be a direct or indirect cause of death.^42^

### Malaria outbreaks.

Dynamic socioeconomic changes and favorable environmental conditions may lead to malaria outbreaks. The predominance of *P. vivax* with an associated high proportion of recurrence illustrates the resilience of this parasite to the usual control measures in the Amazon region. An effort to develop an automated algorithm to detect malaria epidemics identified 338 municipalities presenting outbreaks in 2010.^43^ *Plasmodium vivax* was the main or sole species involved in most localities. Most epidemics were detected in localities where transmission was low or interrupted, demonstrating high vulnerability to reintroduction. In 2014 and 2015, this same system identified 112 and 111 municipalities in an outbreak situation, with alerts sent to local control managers on a weekly basis. The impact of this online real-time system is yet to be evaluated at local level.

Manaus, in the Brazilian Western Amazon region, is among the municipalities with highest incidence of malaria in the country.^3^ The intensification of the malaria control actions led to a reduction from more than 69,000 cases in 2003 to slightly above 10,000 cases in 2012 and 7,300 cases in 2015. This decrease was accompanied by an increase in the proportion of “imported cases,” acquired mostly during work or leisure-related activities in neighboring municipalities (Table 1 ), and indicating a need for changes in control strategies by health authorities.

Although malaria outside the Amazon region is a relatively small problem, it is of considerable public health importance for two main reasons. High mobility of people from areas of malaria transmission (nationally and internationally), results in a need to promptly diagnose and treat new cases. The higher lethality associated with malaria in the extra-Amazon centers underscores the need to improve health systems in this region to provide proper care. The second reason regards the risk of transmission reintroduction, as anophelines are present in most areas, including *An. darlingi* and other species that usually breed in bromeliads of the Atlantic forest.^44^ Proper monitoring and evaluation of innovative strategies should be considered as tools to address non-Amazon malaria transmission risk. An autochthonous outbreak of *P. vivax* occurred in (year) in the southern state of Santa Catarina, where a mass screening and treatment strategy could not eliminate transmission. The population was screened by serology and every positive case was treated, which combined with intensification of case detection and vector control measures led to accelerated reduction in transmission followed by sustainable elimination.^45^

### Prevalence of G6PD deficiency.

The prevalence of G6PD deficiency in an endemic region of the Brazilian Amazon has been estimated to be between 3.4 and 5.6% in adult males, with the majority being of moderate activity associated to the A-genetic variant and a small proportion of severe deficiency associated with the Mediterranean variant.^46^ Although there was strong evidence of the deficient status being protective to malaria infection, individuals with reduced G6PD activity reported a higher incidence of jaundice and blood transfusion when presenting malaria, probably due to primaquine-induced hemolysis. Primaquine has been prescribed in Brazil for decades without prior assessment of G6PD status, posing a potential risk to the small proportion of G6PD-deficient individuals receiving this drug, demonstrated by series of hospitalized patients and fatal cases.^42^,^47^,^48^ A large population survey of G6PD deficiency in the Amazon region is ongoing and should provide valuable information that will help to guide safer policies.

## Malaria Control Interventions

In Brazil, elimination of a species of the *Anopheles gambiae* complex from the northeast region was achieved in the 1940s, and in (year) interruption of transmission in the southern and coastal regions was an important success during the malaria elimination campaign.^3^ However, malaria in the Amazon region is a more difficult scenario due to its low population density and scarcity of reliable transport routes making it operationally difficult to deliver and sustain health care and health preventive measures. One of the mainstays of Brazil's universal health system is decentralization. Although there has been some opposition to decentralize surveillance and control activities, this process begun in 2000. Monitoring and guidance by the NMCP is paramount for the success of malaria control activities as there is broad variation in management of local actions, especially in municipalities that have recently taken these responsibilities.

### Vector control.

Vector-related control measures include routine indoor residual spraying (IRS) and, since 2011, the distribution of LLINs acquired in a Global Fund Project. The coverage of bed nets distribution in malaria-endemic areas increased rapidly; a distribution policy was undertaken by each state. The impact of bed net distribution in regions where *P. vivax* is predominant and relapses are supposed to contribute to a high proportion of cases is still to be properly evaluated. The Ministry of Health issues instructions and provides training on the performance of vector control actions, recommending that IRS is repeated every 4 months and aiming for at least 80% coverage in each locality, what is not usually achieved. A new information system for reporting vector-control activities and entomological indicators is being developed to allow monitoring and assessment of the extent and also the compliance, of LLINs.

In a specific Western Amazon setting, the combination of control measures including the distribution of LLINs and indoor IRS, with the increase of cattle breeding activity was associated with lower densities of *An. darlingi* and reduced incidence of malaria.^17^

### Diagnosis.

There are more than 3,000 microscopy diagnostic units in the Amazon region that performed more than 2.5 million thick blood smears in the year of 2011.^49^ Malaria diagnosis in Brazil is based on both passive and active case detection. Since 2007, most blood slides had been part of active detection (55.9% in 2010), demonstrating the extent of the malaria surveillance and control activities. More than 55% of symptomatic malaria cases are treated within 48 hours of symptoms, which is probably one of the main reasons behind the marked reduction in the proportion of *P. falciparum* cases.

Despite a recent increase in rapid diagnostic test (RDT) distribution and use from 1,486 tests in 2011 growing to 14,655 in 2015, 98% of malaria diagnosis in Brazil is still based on microscopy. There is a high coverage of microscopy-equipped health units, as microscopists are part of the health family teams in the Amazon region. Microscopists are subject to constant training and evaluation, and they also participate in other disease control programs as they are additionally trained to identify trypanosome, filarial, and helminthic infections in many regions. A recent accomplishment has been the inclusion of a malaria result field in the prenatal card to ensure that pregnant women in endemic areas are tested for malaria, as well as RDTs. To identify *P. falciparum* and non-*P. falciparum* infections. Although RDT distribution was initially focused on hard-to-reach areas, its use has been increasing both in the Amazon and non-Amazon regions as a tool in areas where microscopy capacity is lacking and a delay in diagnosing malaria can lead to higher rates of complications.^41^

### Treatment.

Antimalarials in Brazil are free of charge and only available through government facilities. Administration requires a confirmed positive test. The malaria treatment recommendation for *P. vivax* is chloroquine (25 mg/kg divided in 3 days) and primaquine (3.5 mg/kg in 7 or 14 days), apart from pregnant women and children under 6 months of age, which should not receive primaquine.^50^ The Ministry of Health recommends the 7-day regimen in endemic regions to ensure higher compliance with the radical cure treatment. Directly observed therapy is not an established policy in Brazil. There is no recommendation to assess the patient's G6PD deficiency status before prescribing primaquine. The compliance with treatment in a southern Amazon location has been estimated to be around 86%,^51^ but representative assessments and research of risk factors are still necessary.

Both chloroquine and primaquine are produced by Farmaguinhos, the public health Brazilian drug plant, considerably reducing costs. The company has worked on a coated chloroquine pill, to reduce bitterness, and on a coformulated blister of chloroquine with primaquine to facilitate compliance, especially among illiterate populations. There are no specific pediatric formulations of antimalarials, and children have not been routinely included in drug evaluation studies. However, there is some evidence of more pronounced side effects in children in response to treatment.^52^

In Brazil, *P. falciparum* infections are treated with fixed-dose ACTs; addition of single-dose primaquine as a gametocytocide is recommended. Artemeter–lumefantrine is the ACT used in the Amazon region, whereas artesunate–mefloquine is used in nonendemic regions. This decision was made based on a preoccupation of promoting artemisinin resistance due to mefloquine's longer half-life. However, a recent study in the southern amazon demonstrated that ASMQ use for a period of 6 years (between 2006 and 2012) has not led to clinical or molecular evidence of resistance,^53^ supports the decision taken by the Therapeutics Subcommittee of the NMCP of adopting ASMQ for the whole country. Neither chemoprophylaxis nor mass drug administration are common practice in Brazil. Parenteral artemisinins are recommended for severe malaria caused by any parasite species.^50^

### Pharmacovigilance.

There is no systematic antimalarial pharmacovigilance in Brazil, although different reports illustrate its need. Though oral chloroquine is usually safe, it has been demonstrated that pruritus can occur in around 20% of patients being treated for *P. vivax* infection,^54^ with risk of noncompletion of treatment. Adverse events associated with primaquine use, especially hemolysis, are of greater concern^55^ as they can lead to severe complications.^48^ An autopsy series of *P. vivax* patients found severe primaquine-induced hemolysis and associated complications as the main cause of death in two individuals.^42^ The lack of laboratory facilities and tests for G6PD status assessment in the field certainly leads to G6PD deficiency underdiagnosis and underreporting. G6PD status and follow-up of patients receiving primaquine should be a main focus of antimalarial pharmacovigilance system.

### Drug resistance.

Evidence of chloroquine-resistant *P. vivax* in Brazil dates as early as 1999, with the report of a child with parasite resistance to chloroquine and mefloquine.^56^ A study of chloroquine monotherapy efficacy detected 10.1% resistance among 107 cases in the Manaus region,^57^ with a subsequent study evaluating the 28-day efficacy of chloroquine and primaquine finding a recurrence rate of 5.2%.^34^ These findings, all from the Manaus region, support the maintenance of chloroquine as first-line therapy in Brazil. However, a recently completed trial comparing an ACT (artesunate–amodiaquine) to chloroquine following patients for 42 days demonstrated a considerably larger failure rate in the chloroquine arm (under review), raising the need for systematic and representative monitoring.

Primaquine treatment failure has been reported in settings with expected high compliance^31^ even from individuals in transmission-free areas receiving adequate doses.^32^ In a recent multicenter tafenoquine trial, although only six patients from Brazil received primaquine for 14 days the efficacy of this regimen was considerably high, at 83%.^58^ More research on possible causes of primaquine failure is needed, as well as primaquine delivery systems that may be more effective.

## Surveillance and Information System

The Ministry of Health, through its NMCP, is responsible for issuing malaria recommendations and monitoring overall and local trends on malaria incidence, intervening when necessary. The widespread diagnosis and treatment network is also part of the surveillance information system. For each malaria diagnostics procedure case, either positive or negative, is notified, a surveillance form is filled, and data are entered online at the health center or at the corresponding regional post. The supply of antimalarials and laboratory materials is based on the information of number of cases, stimulating prompt reporting of all cases, ensuring reliability of information, and facilitating real-time monitoring of malaria transmission. The information system underwent several changes throughout the years and since 2003, under the acronym SIVEP_Malaria, it is available through an online platform with online data entering and issuing of reports.

There is, however, considerable room for improvement, such as the implementation of a unique identifier number for each person that will allow the routine assessment of repeated malaria episodes per individual, as well as linkage with other health and welfare databases. The automated algorithm to detect epidemics has been recently made available, and is programmed to send a weekly report to locations where the number of cases surpasses a threshold established based on previous years. Its efficacy is yet to be evaluated. As mentioned, an information system specifically designed to register vector-related assessments and control measures is under development and planned to be implemented later in 2016.

### Plans for malaria elimination.

NMCP has recently launched a plan for malaria elimination with a first phase goal of eliminating *P. falciparum* from most of the territory in the next (how many?) years, following World Health Organization's Global Technical Strategy. Although initially focused on *P. falciparum*, this plan will certainly lead to a reduction of *P. vivax* incidence and allow for more specific actions to be directed toward its better management and control. Stratification of areas according to transmission levels and socioenvironmental characteristics is also being pursued to tailor measures to each setting. The development and evaluation of innovative strategies is also a priority, which needs involvement of academia and scientists collaborating with the NMCP to design cost-effective actions.

### Key challenges for *P. vivax* control.

Some of the main challenges for malaria control in Brazil are the maintenance of control actions and investments in areas of low transmission; the use of surveillance data more readily to respond to changing scenarios; the lack of more reliable measures of the contribution of relapses to disease incidence; and the risk of reemergence from neighboring countries with high transmission rates. Specific measures need to be taken to secure that funding and control actions are maintained at local level, requiring collaboration with public partners outside the health sector.

It is usual for local malaria control authorities to conduct microscopic surveys, especially in areas of outbreaks or high transmission. However, this strategy does not detect hypnozoite carriers and it likely misses asymptomatic individuals with low parasitemia. Innovative diagnostic tools and strategies for more effective screening and treatment are therefore urgently needed. Successful experiences should be reviewed to aid in designing and evaluating new strategies. Strategies and tools that can identify the infectious reservoir are paramount, such as the use of more sensitive diagnostic methods that could be deployed in the field. An interest prospect, following the successful experience in Santa Catarina in the 1980s is the use of serosurveys to guide control actions, as this strategy seems to provide reliable estimates of transmission.^59^

## Conclusion

Brazil's recent progress in malaria control can be attributed to a combination of factors, from political commitment to socioeconomic improvements, which leads the country toward new needs including a better understanding and management of *P. vivax* infection. This new stage will require further involvement and boldness of both policy makers and the scientific community for development and application of creative and innovative tools and strategies toward better malaria control and elimination in the Amazon context. The malaria epidemiology history in Brazil and neighboring countries where transmission has increased after some degree malaria control success was achieved reveals a need for better sustained and progressive control actions in this Latin American region.

## Figures and Tables

**Table 1:** Number of malaria episodes diagnosed in the city of Manaus, with the proportion of autochthonous (urban) or “imported cases” in the 2003–2012 period

Year	2003	2004	2005	2006	2007	2008	2009	2010	2011	2012
No. of reported cases	6,9105	5,5661	6,4029	4,0332	4,0116	1,9521	1,6285	1,5548	1,4486	9,645
Autochthonous (%)	15.42	14.83	14.69	15.06	13.07	12.08	91.31	12.32	11.31	10.18
“Imported case” (%)	84.58	85.17	85.31	84.94	86.93	87.92	90.87	87.68	88.69	89.82
